# Research on natal and neonatal teeth in Africa: A systematic scoping review of empirical evidence

**DOI:** 10.1002/hsr2.1242

**Published:** 2023-05-03

**Authors:** Jimoh Amzat, Kehinde K. Kanmodi, Kafayat Aminu, Eyinade A. Egbedina

**Affiliations:** ^1^ Department of Sociology Usmanu Danfodiyo University Sokoto Nigeria; ^2^ Department of Sociology University of Johannesburg Johannesburg South Africa; ^3^ Faculty of Dentistry University of Puthisastra Phnom Penh Cambodia; ^4^ Cephas Health Research Initiative Inc. Ibadan Nigeria; ^5^ School of Health and Life Sciences Teesside University Middlesbrough UK; ^6^ Medical Research Unit Adonai Hospital Karu Nigeria; ^7^ Centre for Child and Adolescent Mental Health University College Hospital Ibadan Nigeria

**Keywords:** Africa, child health, evidence, myths, natal tooth, neonatal tooth, pediatrics, scoping review

## Abstract

**Background and Aims:**

In Africa, natal and neonatal teeth is a culture‐bound phenomenon which is associated with several sociocultural connotations which might affect child survival. Different empirical studies have been conducted in Africa on natal and neonatal teeth; however, no known scoping review has been conducted to map the empirical evidence. This systematic scoping review (SSR) aims to document the available empirical evidence, research gaps, and hotspots on neonatal and natal teeth in Africa.

**Methods:**

The methodology of this SSR was informed by the Joanna Brigg's Institute guidelines for SSRs, and it was reported in accordance with the Preferred Reporting Items for Systematic Reviews and Meta‐Analyses extension for Scoping Reviews. Eleven research databases were systematically searched to scooped out all literatures relevant to the scoping review question, after which they were screened for eligibility based on the review's selection criteria. Only the eligible literatures were included in the review. Data were extracted from the included literatures, after which the extracted data were collated, summarized, and presented as results.

**Results:**

This review included only three journal articles. All the reviewed articles revealed knowledge gaps about natal/neonatal teeth. These articles also found misconceptions around natal/neonatal teeth; for example, nurses' and traditional birth attendants' beliefs about these teeth are contrary to scientifically known facts. Multiple myths associated with natal/neonatal teeth, as indicated in the reviewed studies, point to a poor understanding of the condition.

**Conclusion:**

Neonates and infants are highly vulnerable persons; they require care and safeguarding from dental myths that could threaten their survival. Educative information concerning natal/neonatal teeth should be included in public health education programs to address the observed knowledge gaps among African populations and correct wrong beliefs on neonatal and natal teeth.

## 1 INTRODUCTION

Natal and neonatal teeth (NNT) have been called various names, including congenital teeth, fetal teeth, pre‐deciduous teeth, premature teeth, precociously erupted teeth, and dentitia praecox.^1^, ^2^ A “natal tooth” presents at birth, while a “neonatal tooth” grows or erupts within the first 30 days of life.^2^ The condition is rare as it only occurs about 1 in about 3000−3500 live births.^3^, ^4^, ^5^ The exact cause is unknown, but some studies suggested it could be hereditary or genetic makeup.^6^, ^7^ Natal teeth are three times more common than neonatal teeth.^4^ Irrespective of their incidence, what is common is that they are often attributed to some myths and superstitions, which raise curiosity and concerns, especially among primary caregivers. NNT is often portrayed as dental abnormities requiring treatment.

Leung and Robson^5^ observed that natal teeth often occur in pairs and affect mostly the lower primary central incisors representing early eruption of normal primary (deciduous) dentition. Such teeth resemble normal primary dentition in size and shape but are often smaller and poorly developed with weak or no root formation. The most common concerns for mothers include discomfort during suckling and possible laceration of the mother's breasts, among others.^5^ The interference with breastfeeding, degree of mobility, and cultural beliefs often prompt possible treatment in the form of tooth extraction.^8^, ^9^

Cultural beliefs regarding rare medical cases constitute fundamental concerns among community members. Cultural concern mounts due to the rarity of the condition and lack of knowledge among community members giving room for all sorts of interpretations. A rare medical case, such as NNT, may become a culture‐bound phenomenon with a possibility of a negative connotation which might affect child survival. Hence, children with such rare conditions might face challenges.^10^ In Africa, there is always a strong cultural milieu, practices, and superstitions concerning child health.^11^ This is a systematic scoping review (SSR) of empirical evidence on NNT in Africa. The review aims to document the available empirical evidence, research gaps, and hotspots on NNT in Africa.

## 2 METHODS

### 2.1 Review design

This research was a SSR of empirical evidence. Unlike systematic reviews and meta‐analyses, this review seeks to map all the existing empirical studies on neonatal and natal teeth across various African populations to identify the existing knowledge and gaps in the literature and inform future research on this area of interest.^12^ The review methodology was informed by the prescribed guidelines of the Joanna Brigg's Institute guidelines for SSR, and it was reported in accordance with the Preferred Reporting Items for Systematic Reviews and Meta‐Analyses extension for Scoping Reviews (PRISMA‐ScR) checklist.^12^, ^13^

### 2.2 Research question

This review sets out to address the following research questions:

1. What is the current empirical evidence available on neonatal and natal teeth in Africa?
2. What are the current research gaps concerning neonatal and natal teeth in Africa?
3. Where are the research hotspots on neonatal and natal teeth in Africa?

### 2.3 Study selection

#### 2.3.1 Inclusion criteria

The inclusion of literature into this scoping review was based on the following:

1. Refereed journal papers on neonatal and/or natal teeth.
2. Papers with accessible full text.
3. Papers reporting primary research data.
4. Papers whose language of publication was English.
5. Studies conducted in Africa.
6. Studies adopting qualitative, quantitative, or mixed methods design.

#### 2.3.2 Exclusion criteria

The inclusion of literature into this scoping review was based on the following:

1. Publications on neonatal and/or natal teeth which are not in refereed journals.
2. Papers with inaccessible full text.
3. Papers that were not based on primary research data.
4. Papers whose language of publication was in French, Portuguese, German, or any other non‐English language.
5. Studies conducted outside of Africa.

### 2.4 Literature search strategy

On December 26, 2022, 11 research databases were searched to identify relevant literature on NNT: PubMed; SCOPUS; AMED—The Allied and Complementary Medicine Database (via EBSCO interface); CINAHL Complete (via EBSCO interface); Dentistry and Oral Sciences Source (via EBSCO interface); MEDLINE (via EBSCO interface); SPORTDiscus with Full Text (via EBSCO interface); APA PsycArticles (via EBSCO interface); Psychology and Behavioral Sciences Collection (via EBSCO interface); APA PsycInfo (via EBSCO interface); and CINAHL Ultimate (via EBSCO interface). These database searches were done using Medical Subject Headings (MeSH) terms and Boolean operators (“AND” and “OR”) without year limiters. The MeSH terms and search strings used for the search process are depicted in the Supporting Information: Supplementary file, Tables A1, A2, and A3.

### 2.5 Deduplication

All retrieved literature was imported into the Rayyan software for deduplication.^17^

### 2.6 Selection of studies

The de‐duplicated literature was screened to identify those meeting the review's inclusion criteria. A two‐staged process was adopted for the screening. The screening was performed blindly by two independent researchers using the Rayyan software.^17^ In the first stage, titles and abstracts were screened to exclude irrelevant literature. The remaining literature was then subjected to full‐text screening—the second stage. Based on the selection criteria set for the review, only those eligible articles were included.

### 2.7 Risk of bias assessment

Unlike systematic reviews, the risk of bias assessment of included articles is not a part of scoping review protocols.^12^, ^18^

### 2.8 Data extraction, collation, and charting

Data were extracted from the included articles with a customized data extraction sheet. The obtained data included citation data (authors' names and publication year), study design, study objectives, study's geographical location, studied population, sample size, results, and conclusions. The extracted data were thereafter collated and summarized into themes. Meta‐analysis was not accomplished in this review due to the lack of statistical and methodological homogeneity in the included articles.

## 3 RESULTS

**Figure 1:**
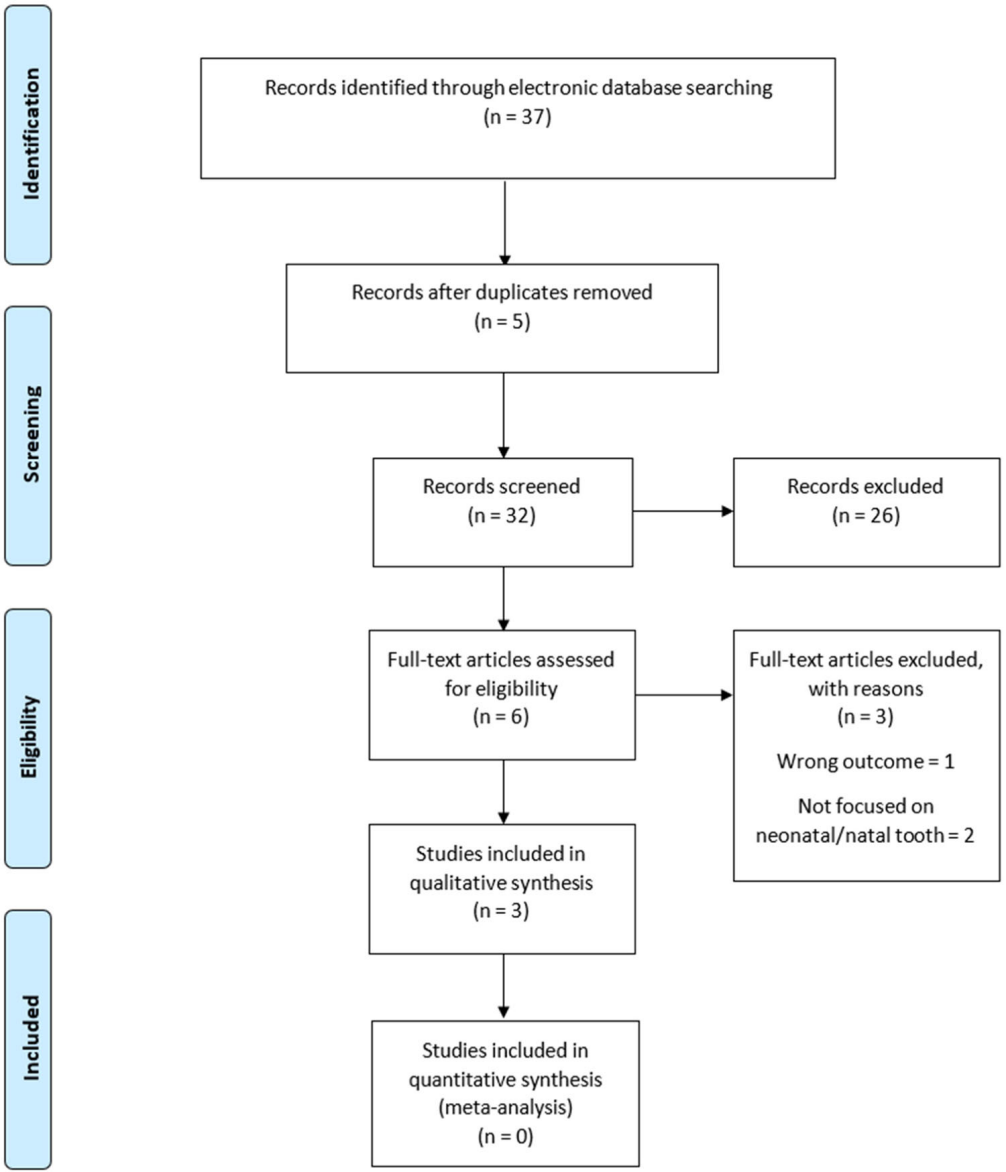
Flow chart of literature search and sorting process.

A total of 37 publications were retrieved from the 11 databases (Table 1). SCOPUS had the highest number (*n* = 25) of retrieved publications. A total of 5 duplicates were identified and removed from the review. From the remaining 32 publications, 26 were excluded after the title and abstract screening. Out of the 6 remaining publications underwent full‐text screening, only 3 were eligible for inclusion in the scoping review (Table 2 and Figure 1).

**Table 1:** Total number of publications retrieved from each database search.

Database	Retrieved publications
PubMed	5
SCOPUS	25
CINAHL Complete (EBSCO interface)	0
CINAHL Ultimate (EBSCO interface)	0
APA PsycInfo (EBSCO interface)	0
APA PsycArticles (EBSCO interface)	0
AMED—The Allied and Complementary Medicine Database (EBSCO interface)	0
MEDLINE (EBSCO interface)	5
SPORTDiscus with Full Text (EBSCO interface)	0
Psychology and Behavioral Sciences (EBSCO interface)	0
Dentistry & Oral Sciences Source (EBSCO interface)	2
Total: 11 databases; 37 publications (including duplicates)	

**Table 2:** List of publications considered for full‐text screening.

Citation	Decision (reason)
Bankole, O. O., & Oke, G. A. (2013). Attitude and beliefs of some nurses in government hospitals in Ibadan, Nigeria to natal/neonatal teeth in infants. *Odonto‐stomatologie tropicale* = *Tropical Dental Journal*, *36*(143), 31–38.	Included
Eigbobo, J. O., Aikins, E. A., & Onyeaso, C. O. (2013). Knowledge of preventive child oral healthcare among expectant mothers in Port Harcourt, Nigeria. *Pediatric Dental Journal*, *23*(1), 1‐7.	Excluded (wrong outcome)
Bankole, O., Taiwo, J., & Nasiru, O. (2012). Attitude and beliefs of traditional birth attendants to prematurely erupted teeth of infants in urban local government areas in Ibadan, Nigeria. *International Quarterly of Community Health Education*, *32*(4), 355‐366.	Included
Bankole, O. O., & Lawal, F. B. (2020). Effectiveness of an oral health education program to improve mothers' awareness of natal teeth: A randomized controlled study. *Pesquisa Brasileira em Odontopediatria e Clínica Integrada*, 20:e0001. https://doi.org/10.1590/pboci.2020.093	Included
Olatosi, O. O., Oyapero, A., Akinwande, K. O., Ayedun, O. S., Aladenika, E. T., & Obe, O. I. (2022). Pattern and prevalence of dental anomalies among a pediatric population in Lagos, Nigeria. *The Nigerian Postgraduate Medical Journal*, *29*(2), 167–172. https://doi.org/10.4103/npmj.npmj_23_22	Excluded (not focused on natal/neonatal tooth)
Chukwumah, N., Azodo, C., & Orikpete, E. (2014). Analysis of tooth mortality among Nigerian children in a tertiary hospital setting. *Annals of Medical and Health Sciences Research*, *4*(3), 345–349. https://doi.org/10.4103/2141-9248.133457	Excluded (not focused on natal/neonatal tooth)

The authors reviewed 3 publications that satisfied the scoping review's selection criteria (see Table 3). Two of the publications utilized cross‐sectional survey design,^14^, ^15^ 1 adopted a randomized control study design.^16^ All three studies reviewed were conducted in Nigeria. However, the cross‐sectional studies were conducted in an urban setting in Ibadan,^14^, ^15^ while the randomized study was domiciled in rural settings, Igboora and Idere.^16^

**Table 3:** Summary of selected publications.

S/N	Author and year of publication	Sample size	Location/country	Objectives	Study design	Population	Intervention	Conclusion
1	Bankole and Oke^14^	380	Ibadan, Oyo state, Nigeria	To assess attitude and beliefs of nurses	Cross‐sectional	Nurses in teaching, general and local government hospitals	None	Nurses' understanding of natal/neonatal teeth was inadequate. Educational intervention can address the wrong beliefs about premature teeth.
2	Bankole, et al.^15^	163	Ibadan, Oyo state, Nigeria	To assess the attitude and beliefs of some Nigerian TBAs to prematurely erupted teeth in infants	Cross‐sectional	Traditional birth attendants	None	Practices of TBAs toward prematurely erupted teeth are alarming and require urgent intervention.
3	Bankole and Lawal^16^	80	Igboora and Idere, Oyo state, Nigeria	To evaluate the effectiveness of an oral health talk aided by a video on improving the awareness of mothers about natal teeth in two rural communities	Cluster randomized controlled study	Mothers	An oral health education program (oral health talk and a Video) on natal teeth	Oral health education program on natal teeth improved mothers' awareness about natal teeth as a normal phenomenon.

Abbreviation: TBA, traditional birth attendants.

Each paper focused on a different population group—mothers,^16^ nurses,^14^ and traditional birth attendants (TBAs).^15^ One of the three papers was an intervention study.^16^ The SSR produced results which are organized thematically, including knowledge and beliefs about natal/neonatal teeth; concerns around natal/neonatal teeth; behavioral dispositions toward natal/neonatal teeth; determinants of beliefs and attitude toward natal/prenatal teeth; and the effects of intervention on knowledge and beliefs about natal/neonatal teeth.

### 3.1 Knowledge and beliefs about natal/neonatal teeth

Understanding of natal/neonatal teeth: All reviewed studies revealed knowledge gaps about natal/neonatal teeth. The studies also found misconceptions about premature teeth as nurses' and TBAs' beliefs about premature teeth are contrary to scientifically known facts.^14^, ^15^ This was assessed through different queries. Only one study conducted among TBAs assessed knowledge of the time of eruption of first milk teeth. The understanding of the time of milk teeth eruption was somewhat low for nearly half of the respondents who mentioned 3−5 (24%), 9−11 (15%), and 12 months upward (6%). The majority, however, put this at 6−8 months (55%).^15^ Some nurses (27.9%) and TBAs (33.1%) had seen prematurely erupted teeth in children.^14^

On the perceived causes of natal/neonatal teeth, both intervention and non‐intervention studies reported misconceptions about the causation of natal/neonatal teeth in children. However, most of the nurses (53.9%) and TBAs (46%) believed prematurely erupted teeth are an individual variation. In addition, nurses believed evil children (16.8%), congenital malformation (8.4%), intake of dangerous drugs (self‐medication) while pregnant (5.5%), and mothers' exposure to other evil children (2.6%) during pregnancy are causative factors.^14^ Similarly, many TBAs mentioned that evil spirits (31.9%) could cause natal/neonatal teeth in children.^15^ Some of the misconceptions common to both groups include the impression that it is caused by prolonged gestational period and mother's violation of taboos.^14^, ^15^

Only one of the three studies assessed beliefs about preventing premature teeth in children. The study reported misconceptions about preventive measures against the occurrence of premature teeth as many TBAs (36.2%) would advise pregnant mothers against violating cultural taboos and some (9.8%) would recommend drinking concoctions to prevent it. However, the majority (54%) believed there were no preventive measures against it.^15^

### 3.2 Concerns around natal/neonatal teeth

A number of myths associated with premature teeth as indicated in the reviewed studies, point at a poor understanding of the condition. Some of the myths on the effects of natal teeth on the child shared by a few nurses (7.4%) and many TBAs (31.3%) are the belief that a child with natal/neonatal teeth will behave strangely.^14^, ^15^ Some nurses thought the child would acquire spiritual authority (12.6%) and experience stigmatization later in life (29.2%). Others indicated that the child would be dull in school (1.3%) and sickly (1.5%).^14^ Furthermore, most TBAs (41.1%) believed the child would develop evil spiritual powers, and some of them (3.1%) thought that the child would be mentally retarded as a result.^15^

In contrast, many nurses and some TBAs expressed a good understanding of the effects of premature teeth on the child. The majority of nurses (43.4%) and some TBAs (24.5%) thought premature teeth would not have any effects on the child.^14^, ^15^ Few nurses revealed that the child would have weak permanent teeth (4.7%) if natal/neonatal teeth erupted.^14^

Reviewed studies reported beliefs on the effects of prematurely erupted teeth on the family, including a manifestation of a curse (nurse‐ 11.8%; TBAs‐ 7.4%). The majority of TBAs (41.1%) and nurses (49.7%) believed the child would cause or be a source of embarrassment to the family.^14^, ^15^ Many TBAs (27%) believed premature teeth would have no effects, while some indicated it was an abomination.^15^

### 3.3 Behavioral dispositions toward natal/neonatal teeth

Generally, the studies reviewed reported positive and negative attitudes toward prematurely erupted teeth. Many nurses (41.3%) revealed that they would be shocked and surprised to see a baby they assisted in delivering with natal teeth, while some (19.2%) would suspect the child was evil. One out of 3 (31.3%) nurses would be shocked to see a child with neonatal teeth.^14^ Likewise, 1 out of 3 TBAs (35.6%) interviewed would be shocked and afraid to see a baby they assisted in delivering with natal teeth, and some (18.4%) would tag the child as weird.^15^ The majority of both TBAs (46%) and nurses (49.2%) would reassure the mother of a child with neonatal teeth.^14^, ^15^

Practices toward prematurely erupted teeth further support evidence of the profound negative attitudes to the phenomenon. Although, signs of positive practices are equally conspicuous such as advising that the child be left alone, as reported by the majority (59.6%) of TBAs and several nurses (33.9%). Nearly half of the nurses would reassure a mother whose child has neonatal teeth.^14^ In addition, approximately 15.3% of nurses would advise the mother to take the child to a pediatrician.^14^, ^15^

Other practices include counseling mothers to extract the prematurely erupted teeth immediately as mentioned by many nurses (39.7%) and TBAs (35.6%). However, we observed some harmful practices along with this advice. The nurses recommended that spiritual cleansing be done before teeth extraction. Similarly, the TBAs who shared this advice wanted the teeth extraction done with or without sacrifice. Furthermore, few TBAs (4.9%) mentioned extreme measures such as asking parents to hide or get rid of the child.^15^

### 3.4 Determinants of beliefs and attitude toward natal/prenatal teeth

Significant determinants of attitude toward prematurely erupted teeth are personal and job‐related factors such as age, education, and years of experience. Nurses who are above 40 years tend to believe such the occurrence of premature teeth would cause embarrassment to the family. Older nurses also are likely to believe that an affected child will experience stigmatization when they grow up. Likewise, immediate extraction of premature teeth would be recommended by nurses older than 26 years.^14^

Likewise, age and educational qualification influenced negative beliefs about premature teeth. TBAs above 50 years and those with primary or no formal education are more likely to associate natal/neonatal teeth condition with evil spirits. Older TBAs tend to believe prematurely erupted teeth are a curse and an abomination in the family. The less educated TBAs are likelier to believe a child with prematurely erupted teeth will bear misfortune compared to the more educated TBAs who did not share this idea.

Long years of practice (>20 years) are significantly associated with beliefs that families with a child with natal/neonatal teeth are cursed and the child would develop evil spiritual powers. This group is also likely to advise mothers to ingest concoctions during pregnancy as precautions.^15^

### 3.5 Effects of intervention on knowledge and beliefs about natal/neonatal teeth

Only one study (intervention) explored knowledge improvement and behavioral change in the study group. Following educational interventions by Bankole and Lawal,^16^ some positive increase in knowledge, attitude, and practice score was evident (34.6% increase). Specifically, significant improvement was recorded in reactions to seeing or assisting in the birth of a child with natal teeth (400% change), causes of natal/neonatal teeth (140% change), advice to mothers of an affected child (61.6% change), positive advice on prevention (118.2% change), possible negative effects of premature teeth on the child (88.9% change).^16^

## 4 DISCUSSION

This SSR focused on empirical research on NNT in Africa. This review identified only three empirical studies on the topic, all conducted in South‐West, Nigeria. Each of these studies focused on population groups (mothers, nurses, and TBAs) who may be directly and indirectly impacted by the phenomenon of natal/neonatal teeth. Besides the peculiarities of each study group, all participants shared a similar culture and worldview enshrined in Yoruba's conception of normal and abnormal phenomena. Africa has a strong heritage of disease conceptions, sometimes based on the mystical etiology of diseases.^19^ The health conception also reflects in the knowledge, practices, including health and illness relating to oral health^20^ (Simangwa et al., 2018^21^, ^22^, ^23^). Beyond the cultural conception, oral is also mediated by several social factors, including gender, age, education, and socioeconomic status.^20^, ^24^, ^25^

In Africa, scientific research on NNT is very low, as two reviewed studies were conducted more than a decade ago. The most recent study by Bankole and Lawal^16^ is an intervention study which did not report details about the community knowledge, attitudes, and practices around premature teeth. The findings are, therefore, not comparable to other publications as the authors primarily focused on observed changes among mothers after the introduced intervention. This low research output on the subject may be due to its infrequency. However, community awareness might modify the response to the condition when it happens. The low research output on the subject is not peculiar to Africa.^26^ also observed the low research outputs regarding NNT, which they claimed require more comprehensive and careful investigation. There have been scientific speculations about the genetic correlates of NNT (Samuel et al.,^27^). Wang et al., on the other hand, observed that the tooth morphology did not have any positive relationships with family history, premature delivery, or the mother's physical condition before delivery.

One of the articles is an intervention study. The intervention involved showing participants a short video on natal teeth, followed by some health talk on oral health delivered by a dentist well‐knowledgeable in the subject. The authors reported positive shifts in the behavioral and knowledge variables tested in the research. The intervention introduced was further effective in addressing misconceptions and myths around neonatal teeth, which have remained stigmatized in the community. The possibility of stigmatizing rare conditions is always very high, including oral conditions or health disparities.^28^ NNT constitute one of the “stigmatized biologies.”^28^ Moeller^29^ reported discrimination concerning dental appearance, disproportionately affecting the socially marginalized. The primary conception is to describe NNT as “abnormal” teeth and hence a source of social concern.

This review shows that, currently, adequate documentation of the prevalence and incidence of NNT in Africa is lacking. This may be associated with the stigma and negative community reaction that the occurrence of NNT tends to attract. It could also point to the fact that mothers of affected children do not present them for treatment at medical facilities. As the TBAs and nurses revealed, extracting strange teeth is believed to require spiritual intervention to avert the negative spiritual consequences on the child and the family. This kind of spiritual interpretation of medical condition is common in Africa,^30^, ^31^, ^32^ which also relates to oral health.^33^, ^34^ Spiritual affiliations might manifest in various forms, which require spiritual cleansing or intervention.

Negative attitudes toward affected child and the family by nurses and TBAs is linked to Yoruba cultural perception of natal teeth phenomenon and the age‐long belief in myths. The TBAs' attitude is also strongly associated with their age, educational status, and long years of practice.^35^ By implication, negative attitudes toward and myths associated with the eruption of premature teeth could expose the child and the family to untold social difficulties. Literature has indicated that in Nigeria, community's attitude to premature teeth is negative due to predominant cultural myths and beliefs.^34^

Comparably, torture, abuse, stigmatization, abandonment, and death remain some of the common experiences of children tagged “witches” in Nigeria, Congo,^36^ Malawi, Ghana, South Africa, Mali, and elsewhere in Africa.^37^ Hence, by implication, associating natal/neonatal teeth with evil spirits, spiritual authority, and other myths could threaten the child's survival and quality of life. Not only about the natal teeth but the cultural misconception of health and illness has also been a significant threat to child survival in many African communities. Hence, myths, misconceptions, and wrong practices are always harmful to newborns and partly explain the high child mortality in sub‐Saharan Africa.^38^ Associating natal/neonatal teeth with evil spirits could lead to child abandonment or low care, including breastfeeding, which could threaten child survival.

Surprisingly, the review could not find any empirical research on natal and neonatal in other African countries except Nigeria. This is a fundamental research gap which should be filled. At least, it is not possible that the NNT only occurs in Nigeria. Due to the similarity of health‐related cultural beliefs across Africa,^19^ it is deducible that the condition could be surrounded by superstitions in other African countries.

## 5 CONCLUSION

This scoping review shows knowledge gaps about natal/neonatal teeth. The knowledge gaps primarily represent misconceptions and lay explanations or beliefs which are at variance with biomedical facts. Aside from community members, especially mothers, some nurses also held such misconceptions. The TBAs also reinforced the misconception that babies with NNT could behave strangely, and be associated with evil and stigmatization. Due to its rarity, NNT is a significant source of social concerns whenever it occurs—it is one of the stigmatized biologies. These beliefs, knowledge gaps, and concerns could threaten child survival. The timing of dental eruption and appearance are essential indicators of childhood oral health. When teeth eruption happens too early in the case of NNT—or too late, it comes with concerns and social implications which should be addressed.

The conclusions of the reviewed studies' conclusion are notable that health education programs should be designed for service providers and members of the community to address the observed knowledge gaps and correct their wrong beliefs about natal/neonatal teeth. For the service providers, this will help them provide correct information/advice to parents and empower them to appropriately manage such situations. Healthcare providers, primary caregivers (especially parents), and community members should be conversant with the implication of NNT and if asymptomatic, such teeth should not be removed. Neonates are fragile and require care, and should be protected from myths and harmful practices threatening child survival. The care provider should fill knowledge gaps through their understanding of cultural apprehension about NNT to ensure infant survival.

## AUTHOR CONTRIBUTIONS

**Jimoh Amzat**: Conceptualization; formal analysis; funding acquisition; investigation; methodology; project administration; resources; supervision; validation; visualization; writing—original draft; writing—review and editing. **Kehinde K. Kanmodi**: Conceptualization; data curation; funding acquisition; investigation; methodology; project administration; resources; software; supervision; validation; visualization; writing—original draft; writing—review and editing. **Kafayat Aminu**: Formal analysis; investigation; validation; writing—original draft. **Eyinade A. Egbedina**: Data curation; investigation; resources.

## CONFLICTS OF INTEREST STATEMENT

Kehinde K. Kanmodi is an Editorial Board member of Health Science Reports and a coauthor of this article. To minimize bias, they were excluded from all editorial decision‐making related to the acceptance of this article for publication. The remaining authors declare no conflict of interest.

## TRANSPARENCY STATEMENT

The lead author Kehinde K. Kanmodi affirms that this manuscript is an honest, accurate, and transparent account of the study being reported; that no important aspects of the study have been omitted; and that any discrepancies from the study as planned (and, if relevant, registered) have been explained.

## Supporting information

Supporting information.

## Data Availability

Data sharing is not applicable to this article as no new data were created or analyzed in this study.
