# Supplementary material for: Research on natal and neonatal teeth in Africa: A systematic scoping review of empirical evidence
Source: Health Sci Rep. 2023 May 3;6(5):e1242. doi: 10.1002/hsr2.1242 (PMC10155507; doi:10.1002/hsr2.1242)
Supplement: Supplementary file 1 — Supporting information. [file HSR2-6-e1242-s001.docx]

**APPENDIX**

**Table A1. Search string for PubMed database search**

| **Tag** | **Subject search** | **Search String** |
| --- | --- | --- |
| #1 | Neonatal/natal tooth | (((neonatal tooth[MeSH Terms]) OR (neonatal teeth[MeSH Terms])) OR (natal tooth[MeSH Terms])) OR (natal teeth[MeSH Terms]) |
| #2 | African countries, dependencies, and territories | (((((((((((((((((((((((((((((((((((((((((((((((((((((((((((Algeria[MeSH Terms]) OR (Angola[MeSH Terms])) OR (Benin[MeSH Terms])) OR (Botswana[MeSH Terms])) OR (burkina faso[MeSH Terms])) OR (burundi[MeSH Terms])) OR (cabo verde[MeSH Terms])) OR (cape verde[MeSH Terms])) OR (cameroon[MeSH Terms])) OR (central african republic[MeSH Terms])) OR (chad[MeSH Terms])) OR (comoros[MeSH Terms])) OR (congo[MeSH Terms])) OR (ivory coast[MeSH Terms])) OR (cote d ivoire[MeSH Terms])) OR (djibouti[MeSH Terms])) OR (democratic republic of congo[MeSH Terms])) OR (egypt[MeSH Terms])) OR (equatorial guinea[MeSH Terms])) OR (eritrea[MeSH Terms])) OR (eswatini[MeSH Terms])) OR (ethiopia[MeSH Terms])) OR (gabon[MeSH Terms])) OR (gambia[MeSH Terms])) OR (ghana[MeSH Terms])) OR (guinea[MeSH Terms])) OR (guinea bissau[MeSH Terms])) OR (kenya[MeSH Terms])) OR (lesotho[MeSH Terms])) OR (liberia[MeSH Terms])) OR (libya[MeSH Terms])) OR (madagascar[MeSH Terms])) OR (malawi[MeSH Terms])) OR (mali[MeSH Terms])) OR (mauritania[MeSH Terms])) OR (mauritius[MeSH Terms])) OR (morocco[MeSH Terms])) OR (mozambique[MeSH Terms])) OR (namibia[MeSH Terms])) OR (niger[MeSH Terms])) OR (nigeria[MeSH Terms])) OR (rwanda[MeSH Terms])) OR (sao tome and principe[MeSH Terms])) OR (senegal[MeSH Terms])) OR (seychelles[MeSH Terms])) OR (sierra leone[MeSH Terms])) OR (somalia[MeSH Terms])) OR (south africa[MeSH Terms])) OR (south sudan[MeSH Terms])) OR (sudan[MeSH Terms])) OR (tanzania[MeSH Terms])) OR (togo[MeSH Terms])) OR (tunisia[MeSH Terms])) OR (uganda[MeSH Terms])) OR (zambia[MeSH Terms])) OR (zimbabwe[MeSH Terms])) OR (reunion[MeSH Terms])) OR (saint helena[MeSH Terms])) OR (western sahara[MeSH Terms])) OR (mayotte[MeSH Terms]) |
| #3 | #1 AND #2 | (#1) AND (#2) |

**Table A2. Search string for SCOPUS database search**

| **Tag** | **Subject search** | **Search String** |
| --- | --- | --- |
| #1 | Neonatal/natal tooth | ( TITLE-ABS-KEY ( natal  AND tooth )  OR  TITLE-ABS-KEY ( natal  AND teeth )  OR  TITLE-ABS-KEY ( neonatal  AND tooth )  OR  TITLE-ABS-KEY ( neonatal  AND teeth ) ) |
| #2 | African countries, dependencies, and territories | ( ( TITLE-ABS-KEY ( angola )  OR  TITLE-ABS-KEY ( benin )  OR  TITLE-ABS-KEY ( botswana )  OR  TITLE-ABS-KEY ( burkina  AND  faso )  OR  TITLE-ABS-KEY ( burundi )  OR  TITLE-ABS-KEY ( cameroon )  OR  TITLE-ABS-KEY ( cabo  AND  verde )  OR  TITLE-ABS-KEY ( cape  AND  verde )  OR  TITLE-ABS-KEY ( central  AND  african  AND  republic )  OR  TITLE-ABS-KEY ( chad )  OR  TITLE-ABS-KEY ( comoros )  OR  TITLE-ABS-KEY ( congo )  OR  TITLE-ABS-KEY ( ivory  AND  coast )  OR  TITLE-ABS-KEY ( democratic  AND  republic  AND  of  AND  congo )  OR  TITLE-ABS-KEY ( djibouti )  OR  TITLE-ABS-KEY ( equatorial  AND  guinea )  OR  TITLE-ABS-KEY ( eritrea )  OR  TITLE-ABS-KEY ( ethiopia )  OR  TITLE-ABS-KEY ( gabon )  OR  TITLE-ABS-KEY ( gambia )  OR  TITLE-ABS-KEY ( ghana )  OR  TITLE-ABS-KEY ( guinea )  OR  TITLE-ABS-KEY ( guinea-bissau )  OR  TITLE-ABS-KEY ( kenya )  OR  TITLE-ABS-KEY ( lesotho )  OR  TITLE-ABS-KEY ( liberia )  OR  TITLE-ABS-KEY ( madagascar )  OR  TITLE-ABS-KEY ( malawi )  OR  TITLE-ABS-KEY ( mali )  OR  TITLE-ABS-KEY ( mauritania )  OR  TITLE-ABS-KEY ( mauritius )  OR  TITLE-ABS-KEY ( mayotte )  OR  TITLE-ABS-KEY ( mozambique )  OR  TITLE-ABS-KEY ( namibia )  OR  TITLE-ABS-KEY ( niger )  OR  TITLE-ABS-KEY ( nigeria )  OR  TITLE-ABS-KEY ( reunion )  OR  TITLE-ABS-KEY ( rwanda )  OR  TITLE-ABS-KEY ( saint  AND  helena )  OR  TITLE-ABS-KEY ( sao  AND  tome  AND  principe )  OR  TITLE-ABS-KEY ( senegal )  OR  TITLE-ABS-KEY ( seychelles )  OR  TITLE-ABS-KEY ( sierra  AND  leone )  OR  TITLE-ABS-KEY ( somalia )  OR  TITLE-ABS-KEY ( south  AND  africa )  OR  TITLE-ABS-KEY ( south  AND  sudan ) ) )  OR  ( ( TITLE-ABS-KEY ( eswatini )  OR  TITLE-ABS-KEY ( togo )  OR  TITLE-ABS-KEY ( uganda )  OR  TITLE-ABS-KEY ( zambia )  OR  TITLE-ABS-KEY ( zimbabwe )  OR  TITLE-ABS-KEY ( egypt )  OR  TITLE-ABS-KEY ( libya )  OR  TITLE-ABS-KEY ( algeria )  OR  TITLE-ABS-KEY ( tunisia )  OR  TITLE-ABS-KEY ( morocco )  OR  TITLE-ABS-KEY ( western  AND  sahara )  OR  TITLE-ABS-KEY ( sudan )  OR  TITLE-ABS-KEY ( tunisia ) ) ) |
| #3 | #1 AND #2 | (#1) AND (#2) |

**Table A3. Search string for other database (AMED – The Allied and Complementary Medicine Database; CINAHL Complete; Dentistry and Oral Sciences Source; MEDLINE; SPORTDiscus with Full Text; APA PsycArticles; Psychology and Behavioral Sciences Collection; APA PsycInfo and CINAHL Ultimate) search via EBSCO interface**

| **Tag** | **Subject search** | **Search String** |
| --- | --- | --- |
| #1 | Neonatal/natal tooth | AB natal tooth OR AB natal teeth OR AB neonatal tooth OR AB neonatal teeth |
| #2 | African countries, dependencies, and territories | AB algeria OR AB angola OR AB benin OR AB botswana OR AB burkina faso OR AB burundi OR AB cape verde OR AB cabo verde OR AB cameroon OR AB central african republic OR AB chad OR AB comoros OR AB congo OR AB cote d'ivoire OR AB ivory coast OR AB djibouti OR AB democratic republic of congo OR AB egypt OR AB equatorial guinea OR AB eritrea OR AB eswatini OR AB ethiopia OR AB gabon OR AB gambia OR AB ghana OR AB guinea OR AB guinea bissau OR AB kenya OR AB lesotho OR AB liberia OR AB libya OR AB madagascar OR AB malawi OR AB mali OR AB mauritania OR AB mauritius OR AB morocco OR AB mozambique OR AB namibia OR AB niger OR AB nigeria OR AB rwanda OR AB ( sao tome and principe ) OR AB senegal OR AB seychelles OR AB sierra leone OR AB somalia OR AB south Africa OR AB south sudan OR AB sudan OR AB tanzania OR AB togo OR AB tunisia OR AB uganda OR AB zambia OR AB zimbabwe OR AB reunion OR AB saint helena OR AB western sahara OR AB mayotte |
| #3 | #1 AND #2 |  |
